# Fear of COVID-19 infection and its relation to depressive and anxiety symptoms among elderly population: online survey

**DOI:** 10.1186/s43045-022-00177-1

**Published:** 2022-01-21

**Authors:** Ola Osama Khalaf, Shaimaa Abdalaleem Abdalgeleel, Nehal Mostafa

**Affiliations:** 1 Department of Psychiatry, Faculty of Medicine, Cairo University, Cairo, Egypt; 2 Department of Biostatistics and Epidemiology, National Cancer Institute, Cairo University, Cairo, Egypt

**Keywords:** COVID-19, Elderly population, Fear, Depression, Anxiety

## Abstract

**Background:**

The pandemic of COVID-19 is considered as one of the major threats that affected all age groups all over the world. Old age group has been highly affected with increased risk of severe health complications that may result in several mental health problems such as anxiety symptoms, depressive symptoms, sleep problems, or any other mental health disorder. Thus the aim of this study is to investigate the fear of COVID-19 infection and its relation to depressive and anxiety symptoms among elderly population during COVID-19 outbreak. A survey was distributed online through social media via a link to people who are 60 years old or above (*N* = 161).

**Results:**

The average score of fear of COVID-19 scale was 17.7 ± 5.4. There was a highly statistically significant correlation between anxiety subscale, depression subscale, and total score of hospital anxiety and depression scale with fear of COVID-19 score.

**Conclusions:**

Participants who were more worried about having the disease developed more symptoms of anxiety and depression during the COVID-19 pandemic. It is necessary to screen the older people for the COVID-19-related fear and accompanying psychological disorders.

## Background

Coronavirus disease of 2019 (COVID-19) is considered as one of the worst pandemics in the recent century [1]. It can be fatal and the number of deaths hugely increased, especially among older adults [2–4].

As people get older, they started to develop many health problems such as high blood pressure (hypertension), diabetes, and stroke [5]. These diseases increase with getting older and considered as one of the poor prognostic factors for COVID-19 and increase the susceptibility to the virus [6, 7].

Moreover, these health problems may coexist and result in several mental health problems such as anxiety symptoms, depressive symptoms, sleep problems, or any other mental health disorder [8].

The prevalence of depression among older people in America is about 8% [9] and 7% worldwide among older adults [10]. Prevalence of sleep disorders among old age people are between 6% and 62.1% depending on the country [11]. Taking into consideration these health problems among older people and their vulnerable health condition, it would be assumed that older people are more likely to be at risk of COVID-19 compared with the younger people [12].

Egypt has been considered among the most affected countries in the Eastern Mediterranean region with 11,228 confirmed cases and 592 mortality cases [13].

As a consequence, many countries, including Egypt, have set a quarantine measures to prevent the spread of COVID-19 among people resulting in a global atmosphere of depression, stress, anxiety, loneliness, and isolation as well as fears of getting infected [14–16].

These factors increased the psychological stress and put the susceptible groups at greater risk for mental health problems specially the elder people [17]. Thus, the aim of the study is to assess the fear of COVID-19 infection among elderly group and to investigate its relation to depressive and anxiety symptoms.

## Methods

### Study design

#### Methods

A cross-sectional study using a convenient sample. A survey was distributed through social media (Facebook and Whatsapp) via a link. The study was conducted from 14 October 2021 to 11 November 2021.

### Study population

Any person who is 60 or above of both genders was included in the study and consenting to participate was a must as it was the first question in the survey, if the participant refused, the survey is closed and no further questions will appear. The survey was written in Arabic.

### Sample size

Based on the study done by Han et al., 2021 mean score of COVID-19 fear scale is 28.66 (8.45). Therefore, a minimum of 119 participants give 80% power and 95% confidence interval. Adding 30% for possibility of using non-parametric test therefore minimum sample size will be 155 [18].

### Tools

Due to the restrictions imposed over the face-to face interaction during the data-collection period, we used Google docs as an online survey constructing tool. Participants were contacted through social networks (Facebook and WhatsApp). The online questionnaire included clarification of the purpose of the study.

#### Socio-demographics parameters

Questions concerning socio-demographic aspects of the participants (e.g., age, gender, educational level) were included in the online survey.

#### Fear of COVID-19 scale (FCV-19S)

##### FCV-19S, Arabic version [19, 20]

It is a 7-item scale that assesses the fear of COVID-19. Each item on the scale is answered using a 5-point, Likert-type scale ranging from 1 (“strongly disagree”) to 5 (“strongly agree”).

#### Hospital Anxiety and Depression Scale (HADS)’

The Arabic version of the Hospital Anxiety and Depression Scale will be used to access anxiety and depression levels of participants [21]*.* The Hospital Anxiety and Depression Scale (HADS) consisted of 14 items, which was one of the most commonly used instruments worldwide for assessing anxiety and depression in clinical patients with physical problems [21].

The HADS consisted of two subscales: anxiety and depression, with seven items each. Participants rated on a 4-point Likert scale (0  =  not at all and 3  =  very much indeed). The scores of each subscale ranged from 0 to 21, with higher scores reflecting higher anxiety and depression. Participants were grouped as follows: 0–7  =  normal, 8–10  =  possible anxiety and depression, 11–21  =  probable anxiety and depression, according to the cut-off values recommended by Zigmond and Snaith [22]. In the current study, unless otherwise stated, anxiety and depression referred to patients whose scores were 8 or greater as rated on the subscales of HADS, and this cut-off was an optimal balance between sensitivity and specificity in most studies [23].

The scale is designed as a screening tool rather than a clinical diagnostic tool and was used in this study as a screening instrument to flag probable anxiety and depression illness.

### Statistical analysis

Data were analyzed using SPSS win statistical package version 17. Numerical data were summarized as means and standard deviations (SD) or medians and ranges as appropriate. Medians were used mainly for skewedness and not normally distributed data. While qualitative data were described as Frequencies and percentages. Comparison between two groups for numerical variables was done using either Student’s *t* test or Mann-Whitney *U* test (non-parametric *t* test) as appropriate.

Comparison between more than two groups for numerical variables was done using ANOVA or non-parametric Kruskal-Wallis as appropriate. Relation between qualitative data was done using chi-square test or Fisher’s exact test as appropriate. Spearman correlation was used to correlate continuous data. A (*P* ≤ 0.05) was considered significant.

### Ethical consideration

The study was approved by the ethical committee (approval no. 2110-504-010). Data were collected anonymously, and after a full explanation of the aim of the study, participants were informed about the target and benefits of the analysis at the beginning of the survey. Participation in the survey was voluntary. Consenting to participate was a must as it was the first question in the survey; if the participant refused, the survey is closed and no further questions will appear.

## Results

The socio-demographic characteristics of the sample are shown in Table 1. Total no. was 161 with a higher number of females than males who answered the form and most responses were answered by age group ranged from 60 to 64 (*n* = 78, 48.4%). Most of the sample were married (*n* = 114, 70.8%), living with spouse and children (*n* = 76, 47.4%), with chronic disease 108 (*n* = 67.1, 67.1%), and were not infected previously with COVID-19 virus (*n* = 78, 48.4%).

**Table 1:** Socio-demographic data among the studied group

Characteristic	Total (***n*** = 161) N/%
**Age groups**	
60–64	78 (48.4)
65–69	53 (32.9)
70–74	19 (11.8)
≥ 75	11 (6.8)
**Sex**	
Male	69 (42.9)
Female	92 (57.1)
**Marital status**	
Single	5 (3.1)
Married	114 (70.8)
Widow	34 (21.1)
Divorced	8 (5.0)
**Education**	
Did not complete primary education	6 (3.7)
Primary/tertiary	14 (8.7)
University	76 (47.2)
Post-graduate	65 (40.4)
**Residence**	
Country side	7 (4.3)
Urban	154 (95.7)
**Living**	
Alone	25 (15.5)
With spouse	32 (19.9)
With spouse and children	76 (47.4)
With children	28 (17.4)
**Work**	
No	95 (59.0)
Yes	66 (41.0)
**Chronic disease**	
No	53 (32.9)
Yes	108 (67.1)
**Infected with COVID-19**	
No	78 (48.4)
Yes	48 (29.8)
Do not know	27 (16.2)
	Mean/S.D
**FCV-19S**	17.7 ± 5.4
	Median/range
**Anxiety subscale of HADS**	5 (1–20)
**Depression subscale of HADS**	7 (3–15)
**Total score of HADS**	12 (5–35)

Average score of fear of COVID-19 scale (FCV-19S) was 17.7 ± 5.4, the relation between FCV-19S and socio-demographic factors were shown in Table 2, and the not educated group scored significantly higher than the highly educated group (*p* = 0.001) on the FCV-19S.

**Table 2:** Relation between fear of COVID-19 scale and socio-demographic factors

Characteristic	Fear of COVID-19 scale	***p*** value
**Total score**	17.7 ± 5.4	
**Age groups**		
60–64	17.5 ± 5.5	
65–69	17.3 ± 5.4	
70–74	17.8 ± 4.9	
≥ 75	20.3 ± 5.6	0.435
**Sex**		
Male	16.7 ± 5.6	
Female	18.4 ± 5.2	0.750
**Marital status**		
Single	14.8 ± 2.8	
Married	17.5 ± 5.6	
Widow	18.9 ± 5.6	
Divorced	16.8 ± 3.5	0.356
**Education**		
Not educated	22.7 ± 7.7	
Primary/tertiary	21.6 ± 5.8	
University	16.5 ± 4.9	
Post-graduate	17.7 ± 5.3	0.001
**Residence**		
Country side	19.6 ± 6.5	
Urban	17.6 ± 5.4	0.353
**Living**		
Alone	18.7 ± 5.3	
With wife	18.1 ± 5.2	
With wife and children	17.1 ± 5.6	
With children	17.9 ± 5.6	0.584
**Work**		
No	18.2 ± 5.7	
Yes	16.9 ± 5.1	0.178
**Chronic disease**		
No	17.1 ± 5.2	
Yes	17.9 ± 5.6	0.311
**Infected with corona**		
No	17.2 ± 6.1	
Yes	18.4 ± 5.2	
Do not know	17.4 ± 4.7	0.497

The current study showed a significant statistical relation between education and fear of COVID-19 as measured by (FCV-19S), where the postgraduate participants were more worried about having COVID-19 infection (*p* = 0.001).

Median score of anxiety subscale, depression subscale and total score of HADS was 5 ranged (1–20), 7 ranged (3–15), and 12 ranged (5–35) respectively.

As regards the relation between anxiety subscale of HADS and socio-demographic factors in Table 3, females were significantly scoring higher than males on anxiety subscale (*p* = 0.016), results showed that they were more prone to anxiety symptoms.

**Table 3:** Relation between anxiety subscale of HADS and socio-demographic factors

Characteristic	Anxiety subscale	***p*** value
**Total score**	5 (1–20)	
**Age groups**		
60–64	4 (1–20)	
65–69	6 (1–16)	
70–74	3 (1–18)	
≥ 75	10 (2–19)	0.136
**Sex**		
Male	3 (1–20)	
Female	6 (1–20)	0.016
**Marital status**		
Single	3 (2–13)	
Married	5 (1–20)	
Widow	6 (1–19)	
Divorced	3 (2–10)	0.146
**Education**		
Not educated	10 (1–20)	
Primary/tertiary	11 (3–20)	
University	4 (1–17)	
Post-graduate	5 (1–16)	< 0.001
**Residence**		
Country side	5 (2–18)	
Urban	5 (1–20)	0.659
**Living**		
Alone	5 (2–19)	
With wife	5 (1–20)	
With wife and children	5 (1–20)	
With children	5 (1–18)	0.864
**Work**		
No	5 (1–20)	
Yes	5 (1–17)	0.466
**Chronic disease**		
No	4 (1–17)	
Yes	5 (1–20)	0.187
**Infected with corona**		
No	4 (1–20)	
Yes	6 (1–20)	
Do not know	7 (1–17)	0.063

Table 4 showed the relation between depression subscale of HADS and socio-demographic factors**,** higher depression scores were among older age group (≥ 75) (*p* = 0.071). Also, females were significantly scoring higher than males on depression subscale (*p* = 0.016), results showed that they were more prone to depressive symptoms. In addition, depression score was significantly greater among less educated group (*p* = 0.013)

**Table 4:** Relation between depression subscale of HADS and socio-demographic factors

Characteristic	Depression subscale	***p*** value
**Total score**	7 (3–15)	
**Age groups**		
60–64	6 (3–15)	
65–69	8 (4–13)	
70–74	8 (3–13)	
≥ 75	9 (4–15)	0.026
**Sex**		
Male	6 (3–15)	
Female	8 (3–15)	0.016
**Marital status**		
Single	7 (3–10)	
Married	7 (3–15)	
Widow	8 (4–15)	
Divorced	7 (4–10)	0.361
**Education**		
Not educated	9 (6–15)	
Primary/tertiary	9 (3–15)	
University	7 (3–13)	
Post-graduate	8 (3–13)	0.013
**Residence**		
Country side	5 (3–13)	
Urban	7 (3–15)	0.659
**Living**		
Alone	8 (3–15)	
With wife	8 (3–15)	
With wife and children	6 (3–13)	0.361
With children	7 (4–13)	
**Work**		
No	7 (3–15)	
Yes	6 (3–13)	0.466
**Chronic disease**		
No	7 (3–13)	
Yes	8 (3–15)	0.187
**Infected with corona**		
No	7 (3–13)	
Yes	7 (3–15)	
Do not know	8 (4–15)	0.767

Moreover, when we studied the relation between total score of HADS and socio-demographic factors, we also found a statistically significant relation between the total score of HADS and female gender (0.007) and education (< 0.001).

Table 5 showed a statistically significant positive correlation between anxiety subscale, depression subscale, and total score of HADS with fear of COVID-19 score (< 0.001, < 0.001, < 0.001), respectively. Participants who were more worried about having the disease developed more symptoms of anxiety and depression.

**Table 5:** Correlation between depression, anxiety subscales of HADS, and the total score with FCV-19S

	FCV-19S	
	Correlation coefficient (***r***)	***p*** value
**Depression subscale**	0.342	< 0.001
**Anxiety subscale**	0.595	< 0.001
**Depression and anxiety score**	0.553	< 0.001

## Discussion

COVID-19 has changed the whole perspective of year 2020 throughout the whole world, affecting various sectors and industries, such as health, education, finance, and manufacturing [24]. On 21 March 2020, some countries like Turkey had announced that a curfew would be imposed for people aged 65 years and older in the scope of COVID-19 measures. Although the number of cases decreased as a result of the measures, the continued announcement of new cases and deaths daily had negative impact on the mental health of individuals and on the society as a whole [25].

This study aimed to assess the prevalence of fear of COVID-19 infection among elderly population and its relation to depressive and anxiety symptoms among elderly population in Egypt. This cross sectional study enrolled 161 participants who responded through online self -administrated questionnaire. Any person who is 60 or above was included in the study.

Most of the participants were females (57.1%), married (70.8%), and with chronic diseases (67.1%). 48.4% of the participants were ranging from 60 to 64 years old and 48.4% of all participants had not been infected with COVID-19.

The current study showed that the total score of fear of COVID-19 scale was 17.7 ± 5.4, females scored higher than males, though the difference was not mounting to a statistically significant but these findings may be contributed to the higher number of stressful and traumatic life experiences in women than in men and the differences in nerve circuits that affect emotional reactivity between males and females ,the hormonal effect (too high or too low estrogen levels) in women and the fact that anxiety disorders are more common in females that males [25] and this was also confirmed when females scored significantly higher on anxiety subscale of HADS in the current study. A Cuban study revealed that women experience fear of COVID-19 more than men, and female gender is a predictor of fear of COVID-19. In the same study, the mean scores of the fear of COVID-19 scale in women were found to be higher than men by 4.00 points [26].

Moreover, as regards the relation between fear of COVID-19 scale and socio-demographics, the current study showed a statistically significant difference between the mean score of fear of COVID-19 scale and education where the post graduate participants showed higher mean score (*p* = 0.001). This finding might be related to being more well educated and having more sufficient knowledge and information regarding the disease transmission and dangerousness which might make them more fearful.

Age was significantly associated with depression subscale of HADS (*p* = 0.026), where the participants who were 75 years old or more had higher mean score of depression. This might be related to the relation between perceived health status of older adults (where most of the participants in the study had chronic diseases) and their mental health which indicates that health status does directly relate with their mental health. Older adults who have poor health status may be more prone to depression and anxiety

These results were concordant with Kemal J et al. 2021 which was done in Ethiopia and showed that females and older age participants (80–91 years old) developed more depressive and anxiety symptoms [27]. The current study was inconsistent with Subjash et al. 2021, which was done in Chandigarh City on 92 elderly participants and also inconsistent with Vahia et al. 2020 which showed that the elderly had lower levels of anxiety and depression and that higher age was associated with lesser psychological impact as well as higher resilience among them. The difference in the results might be related to the differences in the economic status and social support offered by the community and caregivers in different countries as well as the number of participants that were enrolled, also the higher number male participants included in SubJash et al. 2021 study compared to the current study might be one of the causes of inconsistencies between studies as males have more coping mechanisms and can withstand stress more than the females [28, 29].

Also, older adults with poor physical health have challenges with physical activity and in addition to poor lifestyle that may have negative impact on their mental health. Furthermore, people who do not practice physical activity are reportedly two times more likely to exhibit symptoms of depression and anxiety compared to those who perform physical activity regularly as well as the influence of isolation withstand the especially with social connectedness during COVID-19 locking down. The absence of personal social interactions and the lack of physical activities for a long time due to the pandemic have increased the requirement of the society for psychiatric support and aggravated existing psychiatric symptoms **[**8, 25].

As regards correlation between anxiety, depression subscales, and total score of HADS with fear of COVID-19 scale, the current study showed that a high statistically significant positive correlation (< 0.001, < 0.001, < 0.001) respectively. These results were concordant with Ahorsu et al. 2020 in Qazvin which was done on old age group participants and showed fear of COVID-19 can affect the mental health on this group [30]. In another studies by Rossi et al. 2020 done in Italy and Han et al. 2021 in Singapore, it showed a link between anxiety symptoms with both the fear of COVID-19 as well as its association with consequent depressive symptomatology [31, 18].

These results can be explained as fear is a neurological process that help individuals to maintain their integrity in the presence of a threat. It can cause psychiatric disorders like posttraumatic stress disorders, anxiety disorders, depressive disorders, as well as impairment in the ability to suppress a previously learned fear and the capacity to learn safety behaviors.

The relationship between fear of COVID-19 and affective symptoms could be related to catastrophizing, a cognitive error that is commonly associated with anxiety and depression. Catastrophizing is a negative thinking style where one expects the worst possible outcome in a given situation. In COVID-19 pandemic, there was a negative outlook on reality which was accompanied by negative mood and emotion including the high levels of fear. The association among the three variables seems to suggest the presence of a negative reinforcing loop and increased negativity may lead to higher fear levels toward COVID-19, which may in turn contribute to higher levels of depressive and anxiety symptoms [32].

### Limitations

The psychological assessment was based on an online survey and on self-administrated measures. The use of clinical interviews in future studies will give a more comprehensive psychiatric assessment of the participants. Moreover, further studies with a larger randomized sample would help to give more precise data representing the study group.

### Recommendations

The fear of COVID-19 scale, which is a short scale and was applied to participants by social media, revealed that people with higher fear of COVID-19 are in need for psychological help and support. This support can be done through phone calls by professional teams for those who had higher risk. These interventions will decrease the levels of fear in older age groups resulting from an increased level of knowledge and sharing. The World Health Organization (WHO) recommends regular connection with loved ones via phone, e-mail, or video calls; regular routines and schedules such as sleeping, eating, and engaging in favorite home activities; regular and daily activities to maintain mobility; and getting support from family, friends, or neighbors for a minimum of 1-month stock of food and medicines to protect the mental health of older individuals. In China, online counseling services were provided every day of the week by professionals from universities and various medical institutions in many cities during the pandemic [33, 34]. There is a possible plan to provide psychological support to people suffering from the emotional problems due to COVID-19 and other COVID-19-related issues in order to reduce the psychological burden of the pandemic [31].

## Conclusions

Participants who were more worried about having the disease developed more symptoms of anxiety and depression during the COVID-19 pandemic. It is necessary to screen the older people for the COVID-19-related fear and accompanying psychological disorders and to develop appropriate intervention programs for individuals at risk.

## Data Availability

Upon request

## Abbreviations

COVID-19: Corona virus disease of 2019
FCV-19S: Fear of COVID-19 scale
HADS: Hospital Anxiety and Depression Scale
